# Helper Syndrome and Pathological Altruism in nurses – a study in times of the COVID-19 pandemic

**DOI:** 10.3389/fpsyg.2023.1150150

**Published:** 2023-10-12

**Authors:** Victoria E. Maringgele, Martin Scherr, Wolfgang Aichhorn, Andreas K. Kaiser

**Affiliations:** Department of Psychiatry, Psychotherapy and Psychosomatics, Christian Doppler Medical Center, Paracelsus Medical University, Salzburg, Austria

**Keywords:** Pathological Altruism, COVID - 19, health care professionals, Helper Syndrome, nurses, well-being, personality

## Abstract

**Background:**

Pathological Altruism and the concept of Helper Syndrome are comparable. We focused on Schmidbauer’s description because it provides a comprehensive and testable definition. Nevertheless, this concept of Helper Syndrome has not yet been empirically investigated in a sample of helping professionals.

**Aim:**

To investigate whether nurses working with covid-19 patients are more likely to have Helper Syndrome compared with individuals from non-helper professions.

**Methods:**

The online survey took place between April 2021 and February 2022, in urban and rural regions of Salzburg, during the time of the COVID-19 pandemic. Nurses (*n* = 447) and controls (*n* = 295) were compared regarding Helper Syndrome characteristics. To measure characteristics of Helper Syndrome the following questionnaires were used: WHO-Five (WHO-5), selected scales of the Personality, Style and Disorder Inventory (PSSI) and the Freiburg Personality Inventory-Revised (FPI-R), the Alcohol Use Disorders Identification Test (AUDIT). Insecure gender identity and self-assessment of having a Helper Syndrome was measured by a Likert scale.

**Results:**

In both groups, Helper Syndrome was detected (nurses 29.5%, controls 30.5%). Participants with Helper Syndrome showed significant differences in personality styles and traits, namely significantly higher scores for *Foreboding-Schizotypical Personality Style*, *Spontaneous-Borderline Personality Style, Amiable-Histrionic Personality Style, Ambitious-Narcissistic Personality Style, Loyal-Dependent Personality Style, Helpful-Selfless Personality Style, Carefully-Obsessive Personality Style, Optimistic-Rhapsodic Personality Style, Social Orientation, Strain, Emotionality* and lower well-being. The only difference between nurses and controls was that nurses were significantly less open aggressive.

**Conclusion:**

For the first time, we were able to demonstrate Schmidbauer’s concept of Helper Syndrome. According to our data, we found a subgroup of individuals similar to Schmidbauer’s description of Helper Syndrome, but this sample was independent of helping or non-helping profession. These individuals seem to be at higher risk for psychiatric disorders.

## Background

### Definition: Pathological Altruism and Helper Syndrome

This study deals with Pathological Altruism, more precisely with Helper Syndrome, which was introduced in 1977 by the German psychoanalyst Schmidbauer (2018). The concept of Schmidbauer is similar to Pathological Altruism, but it is more comprehensive. By definition, Pathological Altruism is a tendency to promote the welfare of another person, but with negative consequences for the other person or even for oneself (Oakley et al., 2012; Oakley, 2013; Kaufman and Jauk, 2020). Pathological Altruism is also defined by a compulsion to heal, save, and help others (Wong, 2020). Schmidbauer (2018) likewise describes Helper Syndrome also by an increased willingness to help other people and denying one’s own limits. Helper Syndrome (Schmidbauer, 2018) and Pathological Altruism (Kaufman and Jauk, 2020; Wong, 2020) are related to narcissism. According to Schmidbauer, gratitude from the client/patient leads to narcissistic gain and self-esteem is stabilized by sacrificing energy and time for others in need (Schmidbauer, 2018). In addition, helpers with Helper Syndrome find it difficult to express negative feelings such as anger (Schmidbauer, 2018). They show inhibition of direct aggressive behavior. In addition, both Helper Syndrome and Pathological Altruism include dependent behavior toward others (Oakley et al., 2012; Schmidbauer, 2018). Thus, Helper Syndrome describes people who are attracted to helping professions because of a certain personality structure and who perform this profession in a way that leads to symptoms, namely depressive symptoms and pathological alcohol consumption (Schmidbauer, 2018). Also Pathological Altruism cause depressive symptoms (Kaufman and Jauk, 2020).

**Figure 1:**
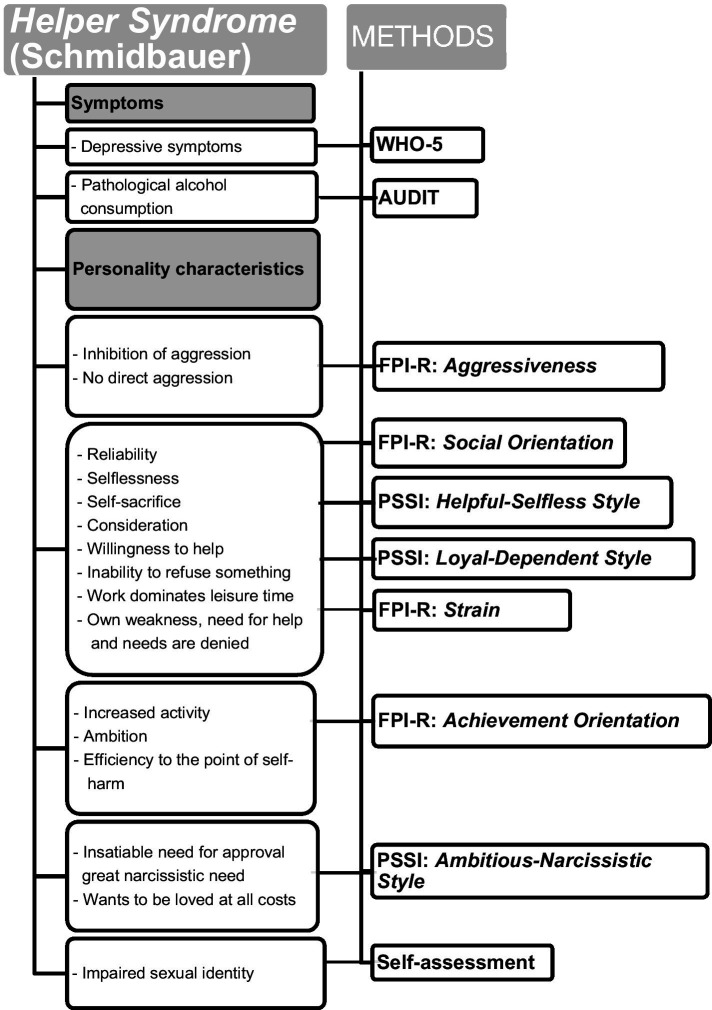
The appearance of *Helper Syndrome* – described by Schmidbauer (2018) – and the survey methods [PSSI, Personality Styles and Disorder Inventory; FPI-R, Freiburg Personality Inventory; AUDIT, Alcohol Use Disorders Identification Test; WHO-5, The World Health Organization – Five Well-Being Index (WHO-5)].

Figure 1 provides a detailed overview of the specific symptoms, personality traits and styles that define Helper Syndrome.

### Helper Syndrome, mental health problems, nursing staff

In addition to Schmidbauer, other studies between 1977 and the present also addressed the mental health of healthcare workers, particularly nurses: One study found that a significant proportion of nurses suffered from symptoms of post-traumatic stress disorder (Cavanaugh et al., 2014). Another study showed that 81 of 561 nurses were in an incipient or advanced burnout process (Schramm, 2016). Other studies have shown an increased risk of burnout among nurses (Cañadas-De la Fuente et al., 2015) and a higher percentage of risky alcohol use among healthcare workers compared to the average population (Romero-Rodríguez et al., 2019). Study results have also shown that a significant proportion of healthcare workers who were responsible for patients with COVID-19 and SARS reported mental health problems, depression, anxiety, and insomnia (Maunder et al., 2006; Lancee et al., 2008; Lai et al., 2020; Muller et al., 2020; Chen et al., 2022). While working conditions undoubtedly affect mental health care professionals (Lancee et al., 2008), mental health problems of health care workers have also been associated with specific personality traits (Pérez-Fuentes et al., 2019). We assumed that during the COVID-19 pandemic, Helper Syndrome would be an additional risk factor for mental health. According to Schmidbauer, Helper Syndrome is more common among health care professionals, triggering depressive symptoms and pathological alcohol consumption.

### Hypotheses and aim of the study

The central question of the study was whether there was a significant difference between nurses and control subjects in terms of certain personality styles and traits as well as symptoms (pathological alcohol consumption, low well-being, insecure gender identity). It is important to emphasize that Schmidbauer first described Helper Syndrome in 1977, and since then more than twenty editions of his book *Helpless Helpers* have been published in German (Schmidbauer, 2018), indicating that many people are interested in this concept. We hypothesized that there are significant differences in Helper Syndrome characteristics between nurses and controls. In his work *Helper Syndrome and Burnout Danger*, published in 2002, Schmidbauer focuses primarily on nursing staff as a typical helping profession, which is why we have chosen the nursing profession as the main group (Schmidbauer, 2002).

## Methods

### Data collection

The survey was conducted using LimeSurvey (2020) and took place between April 2021 and February 2022, in and around Salzburg, during the time of the COVID-19 pandemic. This study was approved by the local ethics committee. Participants received an online link and completed the questionnaires after they had provided written informed consent.

### Participant characteristics

The health care professionals sample (*n* = 447) consisted of graduate frontline nurses with COVID-19 patient contact aged between 20 and 62. The mean age was 39.49 (*SD* 10.89). Managers or nursing staff who were exclusively in teaching positions were excluded. The control group (*n* = 295) consisted of people from other professions (e.g., architects, craftsmen, hairdressers, salesmen, IT, cook, service/waiters and others), aged between 20 and 64 years, without COVID-19 patient contact. The mean age was 39.91 (*SD* 10.94). From this group other “helping professions,” namely teachers, psychotherapists, psychologists, doctors, priests, nuns, educators, secretaries, speech therapists, journalists, and nursing staff were excluded. People in training or retired people were excluded from both groups. The mean age of the health care professionals group and control group did not differ significantly (*p* = 0.61). The following participants were excluded from the sample: 40 participants who did not fit into a category, 42 nursing assistants, 14 nursing staff who did not work with patients, 8 participants who were either too old or too young, 3 nurses who were in training, and 70 participants who worked in other helping-professions. Since we were looking at occupational groups, it was important to us that all participants in the study were employed. No psychiatric disorders were recorded or whether anyone was receiving psychiatric treatment. In total, 742 participants were investigated. The participants did not receive any compensation. It took approximately 25 min to complete the survey.

Table 1 shows sociodemographic characteristics of participants. The Federal Ministry of Social Affairs, Health, Care and Consumer Protection of Austria stated in 2021 that 84% of nursing staff are female (Bundesministerium für Soziales, Gesundheit, Pflege und Konsumentenschutz [The Federal Ministry of Social Affairs, Health, Care and Consumer Protection of Austria states], 2021). With 79% of our sample being female nurses, the gender distribution is representative.

**Table 1:** Sociodemographic characteristic of participants.

	Nurses (*n* = 447)		Controls (*n* = 295)	
	*n*	%	*n*	%
sex				
Women (*n* = 529)	355	79	174	59
Men (*n* = 213)	92	21	121	41
Relationship status				
In relationship	340	76	232	78
Not in relationship	107	24	63	22
Extent of employment				
no indication	2	0	0	0
Part-time	184	42	94	32
Full time	261	58	201	68

Sample size, *n*.

### Measures

Figure 1 shows which psychological methods and scales were used to measure symptoms and personality of Helper Syndrome.

Results of two groups (nurses and controls) were compared using the following psychological tests:

Selected scales of the *Personality, Style and Disorder Inventory* (*PSSI*) and the *Freiburg Personality Inventory, Revised* (*FPI-R*) were used to measure the specific personality structure of Helper Syndrome. The *PSSI* is a self-assessment instrument which measures personality styles. The *PSSI* comprises 140 items assigned to 14 scales (Kuhl and Kazén, 2009). Three personality styles (*Helpful-Selfless Style, Loyal-Dependent Style, Ambitious-Narcissistic Style*) were assigned to Helper Syndrome. The *FPI-R* measures traits of personality. It comprises 138 items and consists of 12 scales (Fahrenberg et al., 2010). Four scales (*Aggressiveness, Social Orientation, Strain, Achievement Orientation*) were used to assess Helper Syndrome. The internal consistency (Cronbach’s alpha) of the scales of the *FPI-R* ranges from *α* = 0.73 to *α* = 0.83. The consistency coefficients (Cronbach’s alpha) of the *PSSI* scales vary from *α* = 0.73 to 0.85. Table 2 shows the descriptions of styles and traits that were important for measuring Helper Syndrome.

**Table 2:** Description of styles and traits that were important to measure Helper Syndrome.

	Description of scale
*Helpful-Selfless Style*	wants to care for someone, good-natured, wants to relieve the suffering of others, has difficulty saying no, focuses more on the needs of others than on their own
*Loyal-Dependent Style*	feels helpless on his own, needs a strong person around, needs a lot of proof of being loved, wants to be cared for, is clingy
*Ambitious-Narcissistic Style*	wants to be special, others should respond to her/his wishes, dreams of great success, wants to be the center of attention, wants to be accepted unconditionally
*Aggressiveness*	Low scores: low-aggressive, reserved, passive-aggressive, inhibited-aggressive, is able to control anger
*Social Orientation*	High scores: feels responsible for other people, is helpful, is motivated to help
*Strain*	High scores: quickly feels overwhelmed by many tasks. Possibly nervousness, exhaustion, exhaustion, stress
*Achievement Orientation*	High scores: Performance orientation, is motivated to perform, efficient, likes to compete

The *WHO-Five Well-Being Index* (*WHO-5*) is a screening questionnaire used to assess psychological well-being. Advantages of the *WHO-5* are its brevity and validity as a screening tool for depression (Topp et al., 2015). Brähler et al. (2007) demonstrated that the German version of the *WHO-5* index has a very good psychometric accuracy. Scores range from 0 to 25, with 0 denoting the lowest well-being and 25 denoting the highest well-being. A score below 13 indicates depression (World Health Organisation-5, 2022).

The *Alcohol Use Disorders Identification Test* (*AUDIT*) is a screening questionnaire to measure unhealthy alcohol consumption. It consists of 10 items (World Health Organisation, 2020). The *AUDIT* is a reliable and valid screening tool for the identification of pathological alcohol consumption (Dybek et al., 2006). Alcohol-related disorder is diagnosed at scores above 7 (Suchtforschungsverbund Baden Württemberg, UKL Freiburg, 2022).

Participants were also asked to assess their gender identity on an adapted *Likert* scale from zero to five (*How masculine do you feel/How feminine do you feel?*)

A second self-assessment on an adapted Likert Scale addressed Helper Syndrome itself (“*I have Helper syndrome. On a scale of 0–5, answer how much this statement applies to you”*). This self-assessment was included to capture how strongly someone assesses themselves as having a Helper Syndrome. This self-assessment does not necessarily have to agree with Schmidbauer’s definition of Helper Syndrome. We were primarily interested in the self-description of the participants.

### Statistical analysis

All analyses were conducted using SPSS v.27 (IBM Corp, 2021). In order to reassess the claims of Schmidbauer, a *t*-test for independent samples was calculated. For the *t*-test (two-sided significance) the *Levene test of equal variance* was used to check for homogeneity of variance. In total, of 31 variables were tested: all personality styles, all personality traits, alcohol consumption, well-being, self-assessments (12 scales of the FPI-R, 14 scales of the PSSI scales, 1 scale of the Audit, 1 scale of the WHO-5, 3 self-assessment scales). Hence, the level of statistical significance was adjusted to *p* = 0.002 (0.05/31) using the Bonferroni correction for multiple hypothesis testing (Bühner and Ziegler, 2009). In order to not ignore the two potential influencing factors of age and sex, a *multiple linear regression* was carried out. Two artificial groups were created: participants of “Helper Syndrome group” rated 4 or 5 on the self-designed scale “I have Helper Syndrome.” The participants of the “Non-Helper Syndrome group” rated 0, 1, 2 or 3. Group differences between “Helper Syndrome group” and “Non-Helper Syndrome group” regarding personality styles, personality traits, femininity, masculinity, well-being, alcohol consumption were also investigated by calculating *t*-tests (for independent samples).

## Results

### Self-assessment *“*I have Helper Syndrome” of nurses and controls

Nurses (*M* = 2.57, SD = 1.49, 95% CI [2.43, 2.71]) and controls (*M* = 2.69, SD = 1.47, 95% CI [2.53, 2.86]) did not differ in their assessment of “I have Helper Syndrome” [t(740) = 1.1, *p* = 0.272]. 29.5% of nurses and 30.5% of controls stated that they have Helper Syndrome.

### Group differences “Helper Syndrome group” and “Non-Helper Syndrome group”

There were no differences in demographic characteristics (gender, occupation, relationship status, extent of employment), except for age (see Table 3). The age of the groups differed significantly (t(740) = 3.44, *p* = 0.001). “Helper Syndrome group” (*M* = 37.57, SD = 11.15, 95% CI [36.09, 39.04]) had a significantly lower mean age than “Non-Helper Syndrome group” (*M* = 40.55, SD = 10.68, 95% CI [39.63, 41.47]).

**Table 3:** Sociodemographic characteristics of “Helper Syndrome group” and “Non-Helper Syndrome group.”

	“Helper Syndrome group” (*n* = 222)		“Non-Helper Syndrome group” (*n* = 520)	
	*n*	%*	*n*	%
Sex				
Women (*n* = 529)	164	74	365	70
Man (*n* = 213)	58	26	155	30
occupation				
Nurses (*n* = 447)	132	59	315	61
Other professions (*n* = 295)	90	41	205	39
Relationship status				
In relationship	170	77	402	77
Not in relationship	52	23	118	23
Extent of employment				
No indication	0	0	2	0
Part-time	81	36	197	38
Full time	141	64	321	62

Sample size, n.

As seen in Table 4, “Helper Syndrome group” (*M* = 13.18, SD = 5.46, 95% CI [12.45, 13.90]) had a significantly (*p* < 0.001) lower mean score in well-being than “Non-Helper Syndrome group” (*M* = 14.93, SD = 5.21, 95% CI [14.48, 15.38]). 43% (*n* = 95, 74 women, 21 men) of participants of “Helper Syndrome group” were under the critical value of 13. In contrast, 27% (*n* = 145, 103 women, 42 men) of “Non-Helper Syndrome group” were under the critical value of 13. As also seen in Table 4, “Helper Syndrome group” (*M* = 4.53, SD = 4.06, 95% CI [3.99, 5.07]) and “Non-Helper Syndrome group” (*M* = 3.72, SD = 3.36, 95% CI [3.43, 4.01]), did not differ significantly in their alcohol consumption (t(740) = 2.819, *p* = 0.005). In these two groups, there were no significant differences in self-assessed masculinity (t(740) = −0.569, *p* = 0.57) and self-assessed femininity (t(740) = 1.752, *p* = 0.08). Group differences between “Helper Syndrome group” and “Non-Helper Syndrome group” regarding personality styles and traits are shown in Table 5.

**Table 4:** Group differences between “Helper Syndrome group” and “Non-Helper Syndrome group” regarding well-being and alcohol consumption.

	*M*	*SD*	95% CI	p*	*d*
*Well-being*				<0.001**	**0.332**
Helper Syndrome group (*n* = 222)	13.18	5.46	[12.45,13.90]		
Non-Helper Syndrome group (*n* = 520)	14.93	5.21	[14.48,15.38]		
*Alcohol consumption*				**0.005**	−0.226
Helper Syndrome group (*n* = 222)	4.53	4.06	[3.99,5.07]		
Non-Helper Syndrome group (*n* = 520)	3.72	3.36	[3.43,4.01]		

^*^Statistical significance level of *p* = 0.002. The values with ^**^ are significant values and because of this it is important that they are in bold. *d*, Cohen’s d (effect size); *n*, Sample size; *M*, Mean; *SD*, Standard deviation.

**Table 5:** Group differences between “Helper Syndrome group” and “Non-Helper Syndrome group” regarding personality styles and traits.

	*M*	*SD*	95% CI	p*	*d*
*Willful-Paranoid PS*				**0.078**	**−0.142**
Helper Syndrome group (*n* = 222)	13.13	4.64	[12.51,13.74]		
Non-Helper Syndrome group (*n* = 520)	12.44	4.93	[12.02,12.87]		
*Reserved-Schizoid PS*				**0.135**	**0.120**
Helper Syndrome group	9.58	4.55	[9.74,10.54]		
Non-Helper Syndrome group	10.14	4.66	[8.98,10.18]		
*Foreboding-Schizotypical PS*				<0.001**	**−0.381**
Helper Syndrome group	12.28	5.45	[11.56,13.00]		
Non-Helper Syndrome group	10.09	5.87	[9.58,10.59]		
*Spontaneous-Borderline PS*				<0.001**	**−0.355**
Helper Syndrome group	*7.62*	*6.07*	[6.81,8.42]		
Non-Helper Syndrome group	*5.69*	*5.14*	[5.24,6.13]		
*Amiable-Histrionic PS*				<0.001**	**−0.383**
Helper Syndrome group	15.66	5.52	[14.93,16.39]		
Non-Helper Syndrome group	13.61	5.29	[13.15,14.07]		
*Ambitious-Narcissistic PS*				<0.001**	**−0.307**
Helper Syndrome group	10.96	4.72	[10.34,11.59]		
Non-Helper Syndrome group	9.62	4.23	[9.25,9.98]		
*Self-Critical-Self-Insecure PS*				**0.008**	**−0.215**
Helper Syndrome group	*11.77*	*5.30*	[11.06,12.47]		
Non-Helper Syndrome group	*10.61*	*5.39*	[10.15,11.08]		
*Loyal-Dependent PS*				<0.001**	**−0.429**
Helper Syndrome group	12.99	5.44	[12.27,13,71]		
Non-Helper Syndrome group	10.77	5.06	[10.34,11.21]		
*Carefully-Obsessive-Compulsive PS*				<0.001**	**−0.371**
Helper Syndrome group	19.16	4.77	[18.53,19.79]		
Non-Helper Syndrome group	17.38	4.79	[16.97,17.80]		
*Critical-negativistic Personality Style*				**0.004**	**−0.233**
Helper Syndrome group	*8.10*	*4.40*	[7.52,8.68]		
Non-Helper Syndrome group	*7.057*	*4.56*	[6.65,7.44]		
*Silent-Depressive Personality Style*				**0.012**	**−0.202**
Helper Syndrome group	*10.19*	*5.31*	[9.49,10.90]		
Non-Helper Syndrome group	*9.16*	*5.02*	[8.73,9.60]		
*Helpful-Selfless PS*				<0.001**	**−1.100**
Helper Syndrome group	17.69	4.34	[17.12,18.26]		
Non-Helper Syndrome group	12.96	4.28	[12.60,13.33]		
*Optimistic-Rhapsodic PS*				<0.001**	**−0.301**
Helper Syndrome group	17.41	5.38	[16.70,18.13]		
Non-Helper Syndrome group	15.82	5.27	[15.36,16.27]		
*Assertive-Antisocial PS*				**0.829**	**−0.017**
Helper Syndrome group	*7.34*	*5.06*	[6.67,8.01]		
Non-Helper Syndrome group	*7.25*	*4.77*	[6.84,7.66]		
*Life satisfaction*				**0.295**	**0.084**
Helper Syndrome group	7.67	2.88	[7.29,8.05]		
Non-Helper Syndrome group	7.92	2.99	[7.66,8.17]		
*Social Orientation*				<0.001**	**−0.468**
Helper Syndrome group	8.27	2.17	[7.98,8.55]		
Non-Helper Syndrome group	7.20	2.32	[7.00,7.40]		
*Achievement Orientation*				**0.124**	**−0.123**
Helper Syndrome group	*7.21*	*2.63*	[6.86,7.56]		
Non-Helper Syndrome group	*6.89*	*2.54*	[6.67,7.11]		
*Inhibitedness*				**0.369**	**−0.072**
Helper Syndrome group	*5.69*	*3.20*	[5.27,6.11]		
Non-Helper Syndrome group	*5.46*	*3.14*	[5.19,5.73]		
*Impulsiveness*				0.002**	**−0.247**
Helper Syndrome group	5.84	3.20	[5.42,6.27]		
Non-Helper Syndrome group	5.07	3.13	[4.80,5.33]		
*Aggressiveness*				**0.091**	**−0.135**
Helper Syndrome group	3.45	2.25	[3.15,3.75]		
Non-Helper Syndrome group	3.15	2.22	[2.96,3.34]		
*Strain*				<0.001**	**−0.456**
Helper Syndrome group	*7.56*	*3.23*	[7.13,7.99]		
Non-Helper Syndrome group	*6.06*	*3.32*	[5.77,6.34]		
*Somatic Complaints*				<0.001**	**−0.350**
Helper Syndrome group	4.13	2.74	[3.76,4.49]		
Non-Helper Syndrome group	3.21	2.55	[2.99,3.43]		
*Health Concern*				**0.509**	**0.053**
Helper Syndrome group	4.86	2.64	[4.52,5.21]		
Non-Helper Syndrome group	*5.00*	*2.62*	[4.78,5.23]		
*Frankness*				**0.583**	**−0.044**
Helper Syndrome group	*6.75*	*2.45*	[6.43,7.08]		
Non-Helper Syndrome group	*6.64*	*2.64*	[6.41,6.87]		
*Extraversion*				**0.006**	**−0.221**
Helper Syndrome group	*7.21*	*3.19*	[6.79,7.63]		
Non-Helper Syndrome group	*6.50*	*3.18*	[6.23,6.78]		
*Emotionality*				<0.001**	**−0.294**
Helper Syndrome group	6.47	3.87	[5.96,6.99]		
Non-Helper Syndrome group	5.35	3.79	[5.02,5.68]		

^*^Statistical significance level of *p* = 0.002. The values with ^**^ are significant values and because of this it is important that they are in bold. *d*, Cohen’s d (effect size); *n*, Sample size; *M*, Mean; *SD*, Standard deviation.

### Personality styles and traits of Helper Syndrome by Schmidbauer

With exception of *Aggressiveness (see below)*, there was no significant difference in Helper Syndrome characteristics between nursing staff and controls. Table 6 shows the group differences between nurses and controls regarding personality characteristics of Helper Syndrome.

**Table 6:** Group differences between nurses and controls regarding personality characteristics of Helper Syndrome (*t*-test for independent samples).

	*M*	*SD*	p*
*Ambitious-Narcissistic Personality Style*			0.064
Nursing staff (*n* = 447)	9.78	4.463	
Controls (*n* = 295)	10.39	4.346	
*Loyal-Dependent Personality Style*			0.256
Nursing staff	11.62	5.467	
Controls	11.17	4.950	
*Helpful-Selfless Personality Style*			0.087
Nursing staff	14.13	4.813	
Controls	14.75	4.781	
*Social Orientation*			0.055
Nursing staff	7.65	2.278	
Controls	7.32	2.385	
*Achievement Orientation*			0.009
Nursing staff	6.78	4.467	
Controls	7.29	2.690	
*Aggressiveness*			0.002**
Nursing staff	3.02	2.157	
Controls	3.56	2.314	
*Strain*			0.798
Nursing staff	6.48	3.325	
Controls	6.55	3.426	

^*^Statistical significance level of *p* = 0.002. The values with ^**^ are significant values and because of this it is important that they are in bold. *d*, Cohen’s d (effect size); *n*, Sample size; *M*, Mean; *SD*, Standard deviation.

#### Aggressiveness

The two groups, nursing staff (*M* = 3.0, *SD* = 2.2, 95% CI [2.82, 3.23]) and controls (*M* = 3.5, *SD* = 2.3, 95% CI [3.30, 3.83]), differed significantly in *Aggressiveness* (*t*(740) = 3.184, *p* = 0.002). The mean value of *Aggressiveness* was significantly higher for controls (*d* = 0,242). In a multiple linear regression, the predictors age and *helper/non-helper* were able to predict *Aggressiveness* significantly: *F*(2.739) = 10.711, *p* < 0.001. The predictor sex was excluded due to insufficient statistical significance (*p* = 0.008). The coefficients *helper/non-helper* (*β* = −0.548; *p* = 0.001) and age (*β* = −0.024; p = 0.001) were significant. There was no multi-collinearity and the residuals were independent. No extreme cases were found among potential outliers. Normally distributed residuals were assumed based on a P–P plot. However, with a multiple determination coefficient (R2) of 0.028 (corrected R2 of 0.026), our model has only a weak explanation of variance.

#### Assertive-Antisocial Personality Style

In contrast to the hypotheses, nurses (*M* = 6.77, *SD* = 4.7, 95% CI [6.34, 7.21]) and controls (*M* = 7.97, *SD* = 5.0, 95% CI [7.46, 8.62]) differed significantly in *Assertive-Antisocial Personality Style* (t(740) = 3.513, *p* < 0.001). The mean value of *Assertive-Antisocial Personality Style* was significantly higher in controls than in nurses. However, only a small effect was found (*d* = 0,264) (Cohen, 1988). Within the framework of a multiple linear regression, the predictors sex, age and *helper/non-helper* were able to predict *Assertive-Antisocial Personality Style* (*F*(3.738) = 17.7, *p* < 0.001). There was no multi-collinearity and the residuals were independent. No extreme cases were found among potential outliers. Normally distributed residuals were assumed based on a P–P plot. The coefficients sex (*β* = 2.096; *p* < 0.001) and age (*β* = −0.057; *p* < 0.001) were significant whereas the coefficient *helper/non-helper* was not (*β* = −8.66; *p* = 0.017). With a multiple determination coefficient (R2) of 0.038 (corrected R2 of 0.034), the model had only a weak explanation of variance. The result of the *multiple regression analysis* indicates that sex is particularly responsible for the result (*t*-test) that nurses differ from controls with regard to this personality style.

### Symptoms of Helper Syndrome by Schmidbauer

Nurses (*M* = 14.32, *SD* = 5.28) and controls (*M* = 14.53, *SD* = 5.39) did not differ significantly regarding well-being/depressive symptoms (*p* = 0.610). Moreover, nurses (*M* = 3.83, *SD* = 3.30) and controls (*M* = 4.17, *SD* = 4.01) did not differ significantly regarding alcohol consumption (*p* = 0.232). Regarding female groups, there were no significant differences in femininity (*p* = 0.658) and masculinity (*p* = 0.101). Also no significant differences in femininity (*p* = 0.776) or masculinity (*p* = 0.398) regarding male groups were found.

## Conclusion

Some of the personality styles and traits defined as characteristic for Helper Syndrome were significantly more expressed in individuals who described themselves as having Helper Syndrome. Especially *Ambitious-Narcissistic Personality Style, Loyal-Dependent Personality Style, Helpful-Selfless Personality Style, Social Orientation, and Strain* were prominent. Furthermore, participants who believe they have Helper Syndrome showed a significant lower well-being, possibly because of their combination of personality styles and traits. Nearly twice as many participants in “Helper Syndrome group” scored lower than the critical score of 13 for well-being, indicating depression in individuals who believe they have Helper Syndrome.

Our results indicate that the helper syndrome theory has flaws. First there was no significant difference in prevalence of Helper Syndrome in nurses and controls. Furthermore, nurses did not show lower well-being and did not consume more alcohol than control subjects. Similarly, there was no evidence of insecure gender identity among nurses of either sex. However, the nurses showed a significantly lower score for Aggressiveness, which is consistent with Schmidbauer (2018). This is confirmed by the finding that nurses suppress the open, direct expression of anger and instead choose forms of passive aggression, such as procrastination, apathy, unresponsiveness, forgetfulness, lack of understanding, or intellectualization (Carol, 1975). Thus, passive aggression is a potential characteristic of nurses, but not people who self-assessed them as having Helper Syndrome. However, we found that individuals who self-assessed themselves as having Helper Syndrome do not have a significantly lower aggression score.

For the first time, we were able to evaluate Schmidbauer’s concept of Helper Syndrome. In synopsis of the studies and the concept of Schmidbauer and the concept of Pathological Altruism, a new clear definition can be derived. According to this, the “new” Helper Syndrome is an occupation-independent personality structure that is narcissistic, schizotypical, Borderline-like, histrionic, carefully-obsessive, rhapsodic, impulsive, somatizing, neurotic, dependent, selfless, socially oriented, and prone to stress and depression. In comparison to the concept of Pathological Altruism, which is defined as behavioral tendency to promote welfare of others with negative consequences for oneself and the other person, our new definition is superior because of its clear correlation to specific personality traits. Future studies should not focus certain profession but on personality traits and styles as potential predictors of mental health problems.

More empirical research is needed to verify these preliminary data.

## Limitations

The voluntary participation of the participants, the one-time testing, the inhomogeneity of the control group, the gender-specific distribution in the samples, and the fact that education was not recorded.

## Ethics statement

The studies involving humans were approved by Ethikkommission Land Salzburg, Austria. The studies were conducted in accordance with the local legislation and institutional requirements. The participants provided their written informed consent to participate in this study.

## Author contributions

VM, MS, and WA: conception or design of the work. VM: data collection. AK and VM: data analysis and interpretation. VM, MS, AK, and WA: drafting the article. WA, MS, and AK: critical revision of the article. VM, WA, MS, and AK: final approval of the version to be published.
